# Peacock’s Bromides

**Published:** 1887-03

**Authors:** 


PEACOCK'S BROMIDES.—W. H. Wolford, M. D., 2633 State street, Chicago, Ill., writes: "I have used Peacock's Bromides in a number of cases with the best results, especially in epilepsy, one case in particular, C. S., a railroad man, having been compelled to quit work on account of the paroxysms coming on every day. After one week's treatment with Peacock's Bromides, the attacks were considerably lessened; now, after two months' treatment, he seems entirely cured and has resumed work. Any case where there is a nerve sedative indicated, I can cheerfully recommend Peacock's Bromides.

